# Real-life Clinical Outcomes of Low-voltage Isolation and Spatiotemporal Dispersion Ablation Strategies for Persistent Atrial Fibrillation

**DOI:** 10.19102/icrm.2023.14103

**Published:** 2023-10-15

**Authors:** Arismendy Nunez Garcia, Brian Liu, Fahd Nadeem, Nikhil Panda, Malcolm Kirk, Cao Thach Tran, Michael Wu

**Affiliations:** 1 Division of Cardiology, Department of Medicine, Lifespan Cardiovascular Institute and Brown University, Providence, RI, USA; 2 Division of Internal Medicine, Lifespan and Brown University, Providence, RI, USA; 3 Division of Cardiology, John Cochran Veteran Affairs Medical Center, St Louis, MO, USA

**Keywords:** Catheter ablation, low-voltage isolation, persistent atrial fibrillation, pulmonary vein isolation, spatiotemporal dispersion

## Abstract

Multiple techniques have been developed in addition to pulmonary vein isolation (PVI) to improve the outcomes of catheter ablation in patients with persistent atrial fibrillation (AF). We sought to evaluate the long-term efficacy of alternative techniques used in our laboratory for the treatment of persistent AF, including spatiotemporal dispersion (SD) and low-voltage isolation (LVI). Consecutive patients with persistent AF who underwent catheter ablation with the studied techniques between July 2016 and December 2019 were included in the study. PVI alone was compared with PVI plus SD and PVI plus LVI in terms of long-term freedom from atrial tachycardia (AT) and AF recurrence. Follow-up data were obtained from clinical records and hospital visits, which included a 7-day Holter monitor and electrocardiograms. The study was approved by the institutional review board of Rhode Island Hospital. A total of 382 patients underwent catheter ablation at our institution during the study period. One hundred seventy-two patients had paroxysmal AF and were excluded from the study. The remaining 210 patients had persistent AF and were included in the study. One hundred and three patients underwent PVI alone, while 48 had the addition of LVI and 59 had SD. Additionally, freedom from AT/AF recurrence at 18 months was 68% in the group that underwent LVI, 49% in the SD group, and 40% in the group that underwent PVI alone (log-rank *P* = .014). Freedom from AF recurrence was 74% in the LVI group, 71% in the SD group, and 43% in the PVI-alone group (log-rank *P* = .002). On multivariate Cox regression, LVI and left atrial size were found to be independent predictors of recurrence (hazard ratio, 0.39; 95% confidence interval, 0.206–0.760; *P* = .005 and hazard ratio, 1.4; 95% confidence interval, 1.105–1.923; *P* = .008, respectively). LVI and SD in addition to PVI were associated with greater freedom from AT/AF recurrence at 18 months compared to PVI alone.

## Introduction

The discovery of the role of pulmonary veins in the pathophysiology of atrial fibrillation (AF) led to an expansion in catheter-based therapies for the treatment of AF.^1^ The cornerstone of the procedure remains electrical isolation of the pulmonary veins (PVI).

The efficacy of PVI for the treatment of AF has been well established. However, the success rate is higher in patients with paroxysmal AF than in those with persistent AF.^2–5^ Given this limitation, several adjunct techniques have been developed to improve the success rate of catheter ablation among patients with persistent AF. Mapping and ablation of areas using complex fractionated atrial electrograms (CFAEs) during AF has been proposed as an adjunctive technique to PVI.^6^ Although early data were very encouraging, subsequent randomized controlled studies failed to replicate the success of early reports.^4,6^ Several reports have demonstrated the role of multipolar catheter mapping to localize potential AF drivers.^7,8^ Seitz et al. reported that electrograms recorded simultaneously by a multipolar catheter displaying spatial and temporal dispersion are indicative of AF drivers. They also reported high termination rates and freedom from AF recurrence following the ablation of areas of spatiotemporal dispersion (SD).^9^

Areas of low voltage and fibrosis in the left atrium have been implicated in the pathogenesis of AF. Studies have shown that left atrial scarring is an independent predictor of procedural failure.^10^ As such, regional fibrosis and low-voltage areas have been used as ablation targets.^11^ Substrate modification and voltage-guided isolation of the posterior wall have been reported with encouraging results.^12,13^

We sought to retrospectively evaluate the efficacy of PVI alone, PVI plus SD, and PVI plus low-voltage isolation (LVI) in the treatment of persistent AF.

## Methods

This study was approved by the institutional review board of Rhode Island Hospital. Given the retrospective nature of the study, informed consent was not obtained.

### Study subjects

A total of 382 patients underwent catheter ablation for AF at our institution during the study period between July 2016 and December 2019. One hundred seventy-two patients had paroxysmal AF and were excluded from the study. The remaining 210 patients had persistent AF and were included in the study. Persistent AF was defined as continuous AF for at least 7 days.

### Procedure

Procedures were performed in the fasting state and under general anesthesia. Single or double transseptal access was used based on the individual operator’s preference. A coronary sinus catheter was used in all cases with a decapolar or duodecapolar catheter. Anticoagulation was used during the procedure with an activated clotting time target of 350–400 s. Three-dimensional electroanatomic geometries of the left atrium and pulmonary veins were reconstructed using either the NavX™ (Abbott, Chicago, IL, USA) or CARTO^®^ (Biosense Webster, Diamond Bar, CA, USA) mapping system. Voltage maps were obtained in all patients; however, these data were only used to define ablation areas in the group that underwent LVI.

As this was a retrospective study, the ablation technique used on each individual patient was based on the operator’s personal practice. Each operator included in the study only used their preferred technique on all their patients with persistent AF, and patient-specific factors did not affect the technique that was chosen for use.

### Pulmonary vein isolation

PVI was performed using a cryoballoon ablation system from Medtronic (Minneapolis, MN, USA) in most of the patients (69%) who underwent PVI alone. Second-generation balloons were used. A minimum of two cryoballoon applications were performed for each vein for a minimum of ≥3 min per application when feasible. Radiofrequency ablation was performed using irrigated contact force-sensing catheters with a targeted ≥5–10 Ω drop in impedance.

Cardioversion was performed at the end of the procedure if patients remained in AF. Demonstration of entrance and exit blocks was used as the procedure endpoint. This group did not receive any additional lesions outside the pulmonary veins.

### Spatiotemporal dispersion

Patients in this group underwent mapping while in AF. If they presented to the laboratory in sinus rhythm, AF was induced with burst pacing with and without isoproterenol. Mapping was performed using a multipolar catheter (PentaRay; Biosense Webster). Areas showing SD were identified according to the criteria described by Seitz et al.^9^

**Figure 1:**
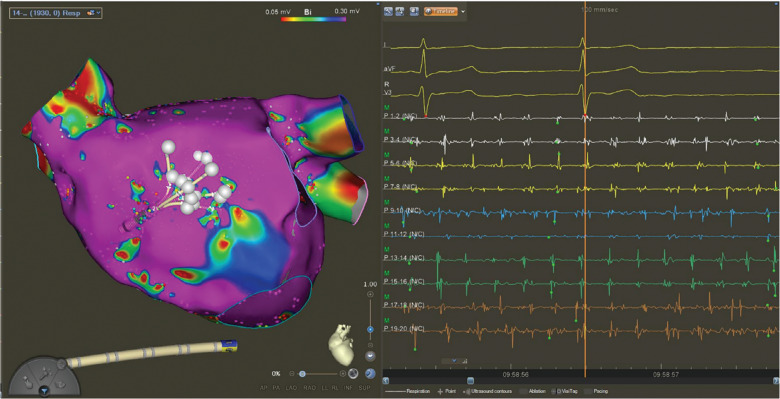
Electroanatomic map of a region showing spatiotemporal dispersion. Electrograms show fractionations and display activation in adjacent bipoles that spread over the entire tachycardia cycle length.

In brief, dispersion areas were defined as clusters of electrograms, either fractionated or non-fractionated, that displayed interelectrode time and space dispersion at a minimum of three adjacent bipoles such that the activation spread over all the AF cycle length. Mapping was performed in both atria ideally to identify all regions of SD **(Figure 1)**.

Following the creation of a complete map, PVI was completed with radiofrequency energy. After PVI completion, all the areas of dispersion were targeted with focal lesions. Isolation of a dispersion area was only performed in the posterior wall when there was widespread dispersion. Areas of dispersion were assessed visually by the operator. No automation or special software was used.

The endpoint was termination of the tachycardia to sinus rhythm or an atrial tachycardia (AT). If termination did not occur despite multiple mapping, sinus rhythm was obtained with cardioversion.

### Low-voltage isolation

A voltage map was created in sinus rhythm according to previously published techniques.^13^

In brief, mapping was performed with a multipolar catheter (PentaRay or Lasso [Biosense Webster]). Low-voltage areas were defined as areas showing a voltage of <0.5 mV.

The low-voltage regions identified with a multipolar catheter were further assessed with detailed point-by-point voltage mapping with an irrigated force-sensing ablation catheter with ≥5 g of contact force.

Pretreatment with an anti-arrhythmic drug and cardioversion was performed several weeks before the procedure to ensure that patients presented to the procedure in sinus rhythm.

If patients presented to the laboratory in AF, cardioversion and/or a bolus of anti-arrhythmics was administered to restore sinus rhythm prior to mapping.

**Figure 2:**
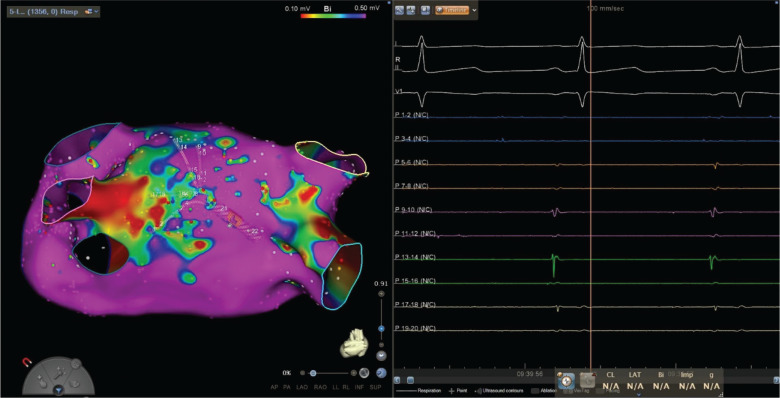
Electroanatomic map and electrograms recorded by the left atrial posterior wall showing low voltage. Low voltage is defined as a measurement of <0.5 mV in sinus rhythm.

Isolation of the low-voltage region was performed based on the operator’s preference. Techniques may involve widening the lesion set for isolating the pulmonary veins to include the low-voltage region or additional linear/focal ablation lesions to isolate a region. In this study, low voltage was mainly performed in the posterior wall, anterior wall, and roof and was not targeted in the floor or in the transseptal region if present (see **Figure 2**). In this group, PVI was completed using radiofrequency energy.

The endpoint was the demonstration of entrance and exit blocks from the isolated regions of low voltage or non-capture if a region was ablated using 10 mA at 2 ms with a contact force of ≥5 g. All patients in this group underwent PVI with the demonstration of entrance and exit blocks.

### Postprocedural care

Oral anticoagulation therapy was continued for ≥3 months in all patients. Thereafter, indication for anticoagulation therapy was based on the patient’s CHA_2_DS_2_-VASc score. The anti-arrhythmic drug was either discontinued or reduced in terms of dosage at 3 months after the procedure if the patient remained in sinus rhythm. If there was frequent recurrence of AF either during or after the blanking period, the anti-arrhythmic drug could be continued or restarted after the blanking period at the operator’s discretion.

Follow-up data were obtained from a chart review of clinic visits, ambulatory electrocardiograms, Holter monitors, and symptom reporting.

The primary endpoint of the study was defined as freedom from AT/AF recurrence among the different ablation strategies after 3 months of the blanking period. Any symptomatic or asymptomatic AT/AF episode that lasted for >30 s was categorized as a recurrence.

### Statistical analysis

Continuous variables were expressed as mean ± standard deviation or median (range) values if indicated. A comparison of the three groups was made using the analysis of variance test. Categorical data were analyzed using a chi-squared (χ^2^) test. Event-free survival was calculated using the Kaplan–Meier method and compared using the log-rank test. Multivariate analysis was performed using the Cox method. Statistical analysis was performed using the SPSS software version 21.0 (IBM Corporation, Armonk, NY, USA).

## Results

### Patient characteristics

A total of 210 consecutive patients with persistent AF met the inclusion criteria and were included in the study. One hundred and three patients underwent PVI alone, 59 underwent PVI plus SD, and 48 underwent PVI plus LVI. Patients who underwent SD were older (68 vs. 64 years, *P* = .011) and had longer continuous AF durations (5 vs. 4 months, *P* = .024) compared to patients in the other two groups.

Otherwise, there were no statistically significant differences in comorbidities, left atrial size, CHA_2_DS_2_-VASc score, left ventricular ejection fraction, or use of anti-arrhythmic drugs among the groups **(Table 1)**.

PVI with demonstration of entrance and exit blocks was achieved in all patients. PVI was performed with cryoablation in 71 (69%) patients and radiofrequency ablation in 32 (31%) patients.

In the group that underwent SD ablation, regions of dispersion were most frequently identified in the posterior wall (70%), and isolation was performed in 38%. Dispersion was also observed in the anterior wall in 60%, roof in 64%, interatrial septum in 38%, and coronary sinus in 13%.

The procedure time was longer for SD (303 ± 90 min) and LVI (293 ± 74 min) compared to in the PVI-only group (242 ± 72 min, *P* < .001). Of note, the procedure time is from time-out to debriefing.

In the LVI group, the lesion set consisted of mainly posterior wall isolation in 64%, roof line isolation in 34%, and superior anterior wall isolation in 15% of the patients **(Table 2)**.

Anti-arrhythmic drugs were used in most of the patients during the blanking period. Amiodarone was used in 39% of the patients who underwent PVI alone, 25% in the SD group, and 38% in the LVI group (*P* = .159).

### Freedom from recurrence

**Figure 3:**
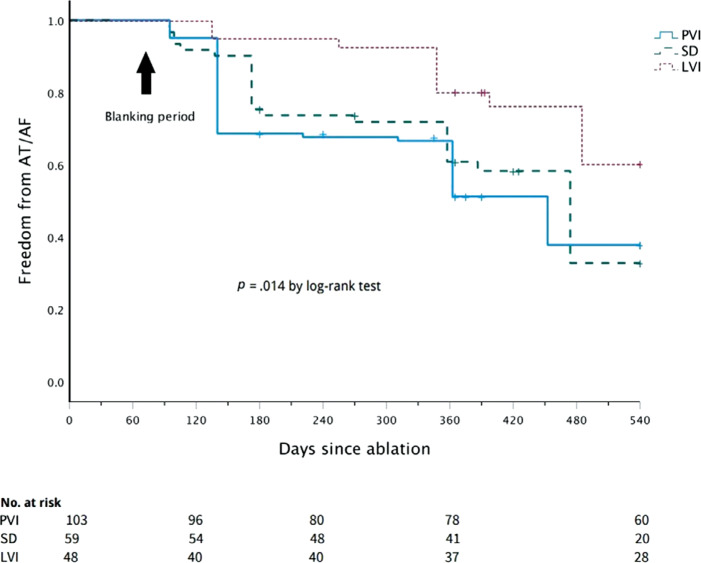
Kaplan–Meier estimates of freedom from recurrence of atrial tachycardia/atrial fibrillation among patients who underwent pulmonary vein isolation alone and the ones who underwent pulmonary vein isolation with the addition of low-voltage isolation and spatiotemporal dispersion. *Abbreviations:* LVI, low-voltage isolation; PVI, pulmonary vein isolation; SD, spatiotemporal dispersion.

**Figure 4:**
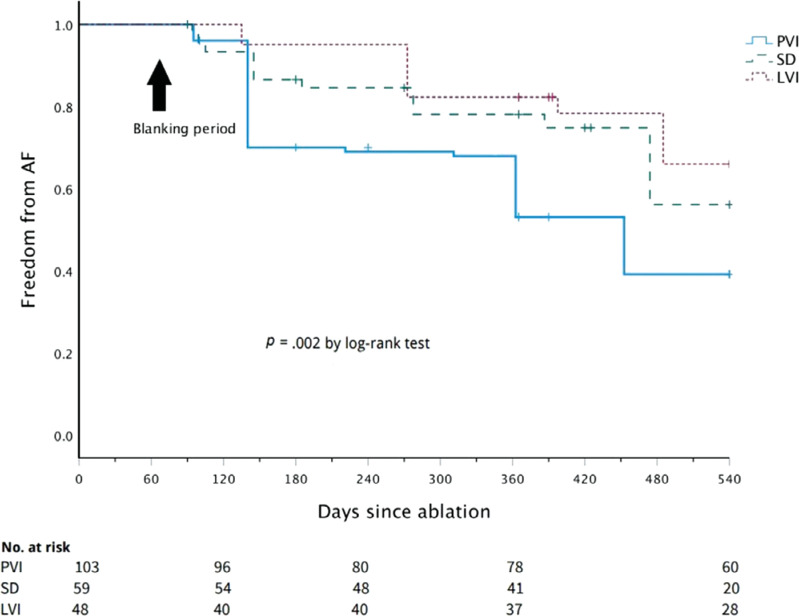
Kaplan–Meier estimates of freedom from recurrence of atrial fibrillation among patients who underwent pulmonary vein isolation alone and the ones who underwent pulmonary vein isolation with the addition of low-voltage isolation and spatiotemporal dispersion. *Abbreviations:* LVI, low-voltage isolation; PVI, pulmonary vein isolation; SD, spatiotemporal dispersion.

Following a 3-month blanking period, freedom from AT/AF recurrence at 18 months was 67% in the PVI plus LVI group, 49% in the PVI plus SD group, and 40% in the PVI-alone group (log-rank *P* = .014) **(Figure 3)**. Separately, freedom from AF recurrence was 74% in the LVI group, 71% in the SD group, and 43% in the PVI-alone group (log-rank *P* = .002) **(Figure 4)**.

On multivariate analysis using Cox regression, freedom from recurrence of AF/AT was independently associated with PVI plus LVI (hazard ratio, 0.39; 95% confidence interval, 0.206–0.760; *P* = .005) and left atrial size (hazard ratio, 1.4; 95% confidence interval, 1.105–1.923; *P* = .008) after correcting for the CHA_2_DS_2_-VASc score, sleep apnea, acute termination, and duration of AF.

### Secondary analysis

#### Left atrial size

Left atrial size was found to be an independent predictor of outcome in this study. Freedom from AT/AF recurrence in patients with a left atrial size of ≤5 cm was 76% in the LVI group, 59% in the SD group, and 47% in the PVI group, respectively. Freedom from AF recurrence in this cohort was 80% in the LVI group, 79% in the SD group, and 50% in the PVI group (log-rank *P* = .021).

In patients with a left atrial size >5 cm, freedom from AT/AF recurrence was 57% in the LVI group, 46% in the SD group, and 37% in the PVI-alone group (log-rank *P* = .262). Freedom from AF recurrence in this cohort was 62% in the LVI group, 73% in the SD group, and 41% in the PVI-alone group (log-rank *P* = .054).

#### Cryoablation versus radiofrequency ablation

In the group that underwent PVI alone, cryoablation was performed in 71 (69%) patients and radiofrequency ablation was completed in 32 (31%) patients. The rate of freedom from AT/AF recurrence was numerically higher for radiofrequency ablation compared to that for cryoablation (54% vs. 49%); however, it did not reach statistical significance (log-rank *P* = .18).

#### Atrial fibrillation termination

AF termination during the procedure was observed in 64% of patients who underwent SD, 0% of patients who underwent LVI, and 2% of patients who underwent PVI alone (*P* < .001). Termination was associated with freedom from AF recurrence (χ^2^ = 5.1, *df* = 1, *P* = .02) but not with freedom from AT/AF recurrence (χ^2^ = 1.7, *df* = 1, *P* = .19).

#### Complications

There was one pericardial effusion that was successfully drained after completion of the case in a patient who underwent SD ablation.

## Discussion

The ideal ablation strategy for patients with persistent AF remains elusive. Randomized controlled trials have not demonstrated consistent improvement in outcomes when a strategy targeting CFAEs or the addition of linear ablation lesions was used compared to PVI alone.^4^ However, since publication of the Substrate and Trigger Ablation for Reduction of Atrial Fibrillation Trial Part II (STAR AF II), additional ablation strategies have been developed and have shown some promise. Two such strategies are the isolation of low-voltage areas and targeting electrograms showing SD.

In this study, we retrospectively evaluated the efficacy of SD and LVI in addition to PVI for persistent AF. The results showed an improvement in freedom from AF and AT recurrence at 18 months for both SD and LVI compared to PVI alone. However, LVI showed a higher rate of freedom from recurrence of AT/AF when compared to the other two techniques.

These results are consistent with other observational reports that showed comparable freedoms from recurrence in patients who underwent LVI.^13–16^ A recent randomized trial also showed improvement in outcomes using this technique.^17^

Our results differ, however, from those of a randomized trial that used magnetic resonance imaging to identify and guide scar ablation.^18^ However, there are some key differences between the techniques used between the two cohorts. First, patients included in the DECAAF II trial did not undergo complete isolation of the regions of low voltage but rather homogenization. Second, the scar-determination method was different (magnetic resonance imaging vs. electroanatomic mapping).

We observed a lower termination rate and freedom from recurrence in the group that underwent SD when compared to the findings reported by Seitz et al. Some potential reasons for the different results include that all the patients in our cohort underwent the addition of PVI, which increased the procedure time. As a result, cardioversion was performed in cases where, in the original cohort, additional ablation and mapping would have been performed. In addition, the left atrial size in our patient population was larger at 5.1 versus 4.5 cm in the French cohort.^9^

Another consideration is the presence of interoperator variability in SD signal interpretation, which could have led to the observed differences in procedure results compared to the original cohort. A recent report using an artificial intelligence algorithm showed improved interoperator variability and may offer a solution to this issue; however, additional studies are needed to confirm their results.^19^ Lastly, a majority of patients in the SD group did not undergo right atrial SD mapping and ablation.

Procedure times were longer in patients who underwent both SD and LVI ablation, which is expected as both groups require more time in mapping and ablation.

AT recurrence was more common in patients who underwent SD ablation. The high occurrence of AT with the spatiotemporal group could be secondary to a pro-arrhythmic effect of isolated lesions not being anchored to zones of electrically silent tissue. This contrasts with the LVI and PVI strategies that used entrance and exit blocks from isolated areas as part of the procedure endpoints. Other reasons could be due to difficulty in achieving adequate ablation lesions in the interatrial septal and coronary sinus regions.

Another important consideration was that there was some heterogeneity in the studied groups and patients in the spatiotemporal group compared to the other two groups were older and had continuous AF for a longer time. In addition, patients in the SD group had a trend toward a larger left atrium that did not meet statistical significance, which raises the possibility that the inferior results in this group could be due to a sicker population.

Anti-arrhythmic drugs were used in a non-negligible number of patients post-ablation. During the blanking period, amiodarone was used in 39% of patients who underwent PVI alone, 25% of patients who had the addition of SD, and 38% of patients who underwent LVI (*P* = .159). The difference in anti-arrhythmic medication use did not reach statistical significance, which would likely not have explained the differences observed among the treatment groups.

### Limitations

This was a non-controlled, observational, retrospective study conducted at a single center. The procedures were performed by different operators, which could have impacted the different procedural outcomes. The techniques used were consistent with each operator’s practice rather than the patient’s clinical characteristics.

The outcomes were measured by reviewing clinical notes, electrocardiograms, and Holter monitors during follow-up, which may have overestimated the success rate in all the three groups. However, because the same follow-up method was used in all the three groups, the differences observed should remain unaffected.

PVI was performed with a cryoballoon and radiofrequency ablation catheter in the different groups, which could have accounted for some of the differences between the groups. However, regardless of the energy source used, PVI was documented in all the patients. In addition, in our own cohort, there was no statistically significant difference in recurrence rate between the group of patients who underwent cryoballoon ablation and those who underwent radiofrequency ablation.

## Conclusions

In conclusion, LVI added to PVI was associated with an improved freedom from AT/AF recurrence at 18 months compared to PVI alone. Ablation of regions showing SD was associated with a high termination rate of AF and a statistically significant improvement in freedom from AF recurrence but not AT/AF recurrence at 18 months compared to PVI alone.

Both LVI and SD ablation demonstrated some improvement in freedom from AT/AF recurrence at 18 months compared to PVI alone for persistent AF. However, procedure times were significantly longer with both techniques. The value of the addition of these techniques to standard PVI merits further study in a larger population.

## Figures and Tables

**Table 1:** Patient Characteristics

Variable	PVI (n = 103)	SD (n = 59)	LVI (n = 48)	*P* Value
Age, years	64 ± 9.6	68 ± 8.3	64 ± 9.4	.011
Male sex, %	78%	68%	67%	.261
AF duration, months	3.8 ± 3.6	5.4 ± 4.4	3.8 ± 3	.024
Hypertension, %	76	81	78	.709
Diabetes, %	24	18	20	.681
OSA	30	42	37	.299
Heart failure	15	20	27	.245
Amiodarone	39	25	38	.159
Sotalol	14.5	18	4	.073
Dofetilide	15.5	10.1	24	.128
Flecainide	6.7	5	2	.467
LA diameter, cm	4.9 ± 0.83	5.1 ± 0.85	4.9 ± 0.72	.096
Left ventricular ejection fraction, %	52 ± 12	52 ± 12	53 ± 10	.89
CHA_2_DS_2_-VASc score, points	2.5 ± 1.5	2.7 ± 1.4	2.4 ± 1.6	.479
Total procedural duration, min (time-out to debriefing)	242 ± 72	303 ± 90	293 ± 74	<.001
Termination, %	2%^a^	64%	0%^b^	<.001

*Abbreviations:* AF, atrial fibrillation; LA, left atrium; LVI, low-voltage isolation; OSA, obstructive sleep apnea; PVI, pulmonary vein isolation; SD, spatiotemporal dispersion. ^a^Among patients who were in AF at the time of the procedure. ^b^Mapped in sinus rhythm.

**Table 2:** Targeted Regions Based on Technique

Technique	Targeted Regions
PVI	Pulmonary veins
PVI plus SD	Pulmonary veins, posterior wall, anterior wall, roof, interatrial septum, and coronary sinus
PVI plus LVI	Pulmonary veins, poterior wall, roof, and superior anterior wall

*Abbreviations:* LVI, low-voltage isolation; PVI, pulmonary vein isolation; SD, spatiotemporal dispersion.
